# α-Synuclein: A Multifunctional Player in Exocytosis, Endocytosis, and Vesicle Recycling

**DOI:** 10.3389/fnins.2019.00028

**Published:** 2019-01-28

**Authors:** Mingzhu Huang, Bianbian Wang, Xiaopeng Li, Chongluo Fu, Changhe Wang, Xinjiang Kang

**Affiliations:** ^1^School of Life Sciences, Liaocheng University, Liaocheng, China; ^2^Center for Mitochondrial Biology and Medicine, The Key Laboratory of Biomedical Information Engineering of Ministry of Education, School of Life Science and Technology and Frontier Institute of Science and Technology, Xi’an Jiaotong University, Xi’an, China; ^3^Key Laboratory of Medical Electrophysiology of Ministry of Education and Medical Electrophysiological Key Laboratory of Sichuan Province, Institute of Cardiovascular Research, Southwest Medical University, Luzhou, China

**Keywords:** α-synuclein, Parkinson’s disease, exocytosis, endocytosis, vesicle recycling

## Abstract

α-synuclein (α-Syn) is a presynaptic enriched protein involved in the pathogenesis of Parkinson’s disease. However, the physiological roles of α-Syn remain poorly understood. Recent studies have indicated a critical role of α-Syn in the sensing and generation of membrane curvature during vesicular exocytosis and endocytosis. It has been known to modulate the assembly of SNARE complex during exocytosis including vesicle docking, priming and fusion steps. Growing evidence suggests that α-Syn also plays critical roles in the endocytosis of synaptic vesicles. It also modulates the availability of releasable vesicles by promoting synaptic vesicles clustering. Here, we provide an overview of recent progresses in understanding the function of α-Syn in the regulation of exocytosis, endocytosis, and vesicle recycling under physiological and pathological conditions.

## Introduction

α-synuclein (α-Syn), encoded by *SNCA1/PARK1* gene, consists of 140 amino acids that structurally contains three major domains: an amphipathic helix-containing N-terminal membrane binding domain (residues 1–60), a hydrophobic non-amyloid-beta component (NAC) domain (residues 61–95) relevant to α-Syn aggregation, and a hydrophilic C-terminal domain (residues 96–140) with chaperone-like activity (Recchia et al., 2004). α-Syn is an intrinsically disordered monomeric protein in aqueous solution (Fauvet et al., 2012; Theillet et al., 2016). However, the N-terminal domain of α-Syn forms an amphipathic α-helix following the interaction with negatively charged membrane lipids (Davidson et al., 1998; Jao et al., 2008; Maltsev et al., 2013), while the C-terminal domain is still unstructured (Bodner et al., 2009). In cytosol, α-Syn monomers stepwise form into amyloid fibrils (Conway et al., 2000; Nonaka et al., 2010; Giehm et al., 2011; Horvath et al., 2012; Lashuel et al., 2013). However, recent studies reveal that N-terminally acetylated α-Syn is preserved in disordered monomer conditions against oligomerization in non-neuronal and neuronal cells under physiological conditions (Theillet et al., 2016; Medeiros et al., 2017). Whereas, the membrane-bound monomers undergo conformational change to form β-sheet-rich intermediates (protofibrils) upon accumulation, and then self-associate to ring-like oligomers or amyloid fibrils on the membrane (Wang et al., 2016). Both of cytosol and membrane-bound ring-like oligomers can form transmembrane pores which disrupt the integrity of the membrane, intracellular calcium homeostasis and signaling (Lashuel et al., 2002; Lashuel et al., 2013). Lewy bodies are intracellular inclusions mainly composed by α-Syn fibrils.

α-synuclein is abundant in presynapse and interacts with synaptic vesicles (SVs) to modulate vesicle recycling physiologically (Maroteaux et al., 1988; Jensen et al., 1998). α-Syn also plays critical roles in PD pathogenesis. Both duplication/triplication (Singleton et al., 2003; Ibanez et al., 2004) and point mutations (e.g., A30P, E46K, and A53T) in *SNCA1* (Polymeropoulos et al., 1997; Kruger et al., 1998; Zarranz et al., 2004) are associated with autosomal dominate familial PD. Among the PD-linked mutations, A53T is located at the dimer interface of the fibril and E46K is related to the stabilization of the protofilament. The structure of fibril carrying both mutations is distinct from that of wild-type, such as reduced helical periodicity. In addition, the E46K fibril shows a right-handed chirality that is distinct from the left-handed chirality of the wild-type and A53T fibrils (Li et al., 2018). Recent study also indicates that peptides derived from α-Syn act as antigenic epitopes presented by major histocompatibility complex protein to be recognized by T cells in PD patients and hints that PD may be a kind of autoimmune disease (Sulzer et al., 2017). Some studies show that oligomers (Fusco et al., 2017; Mor et al., 2017), protofibrils (Stefanis, 2012), intermediates and fibrils (Peelaerts et al., 2015; Wong and Krainc, 2017; Li et al., 2018) of α-Syn are toxic agents, of note, α-Syn fibrils are transmissible to induce the aggregation of endogenous α-Syn in primary neurons (Li et al., 2018). Thus, the toxic gain-of-function effect of α-Syn mutants may mediate PD pathogenesis (Tsika et al., 2010; Colla et al., 2012). Whereas, recent evidence shows that mature fibrils are non-toxic, and even the toxicity of the oligomers depends on the amount of β-sheet content in the rigid regions of them (Bartels et al., 2011; Cremades et al., 2012; Fusco et al., 2017). Here we mainly focus on the physiological and pathological roles of α-Syn in membrane remodeling and vesicle recycling at nerve terminals.

## α-Syn Is a Curvature Sensing and Deforming Protein

α-synuclein, a curvature sensing and generating protein (Varkey et al., 2010; Braun et al., 2012; Shen et al., 2012; Westphal and Chandra, 2013), functions as an amphipathic lipid packing sensor (ALPS). It is reported that the binding affinity of α-Syn with small unilamellar vesicles is 15-fold higher than that with large unilamellar vesicles, suggesting that α-Syn may be a curvature-sensing protein (Middleton and Rhoades, 2010). Alternatively, Hatzakis and coworkers propose that the intrinsic curvature selective binding is mediated by higher density of binding sites on highly curved membranes (Hatzakis et al., 2009; Bhatia et al., 2010). The amphipathic N-terminus of α-Syn is composed of hydrophobic and polar amino acids, which are segregated at opposite sides of the α-helix. These helices are arranged parallel to the membrane bilayer, with their hydrophobic faces inserting into the outer leaflet of the bilayer, creating the asymmetry curvature of the two leaflets of the bilayer and thus causing the membrane curvature (Shen et al., 2012; Westphal and Chandra, 2013). Braun et al. (2012) also report that α-Syn induces both positive mean curvature and negative gaussian curvature in Membranes. Compared to α-Syn, most of the other ALPS proteins, e.g., endophilin and amphiphysin, are structurally different, containing both highly curved scaffolding BAR (Bin-amphiphysin/rvs) domain (Peter et al., 2004; McMahon and Gallop, 2005; Frost et al., 2009; Mim et al., 2012) and membrane-inserting N-terminal amphipathic helix (McMahon and Gallop, 2005; Campelo et al., 2008) for membrane curvature. It is reported that α-Syn tubulation activity (a form of membrane curvature) is about 2.5-fold less than that of endophilin A1, probably attributed to the fact that α-Syn only wedges the amphipathic helix into the membrane to induce membrane curvature without the assistance of BAR domain (Westphal and Chandra, 2013). Moreover, not all of the α-Syn species are involved in the generation of membrane curvature, and only monomeric α-Syn can take the activities. Pathologically, regarding the PD-related α-Syn mutations, A30P mutant that shows decreased phospholipid binding activity is deficient in membrane bending (Westphal and Chandra, 2013). Whereas E46K and A53T mutations, which have increased or comparable phospholipid binding activity, show similar membrane tubulation activities to that of wild type α-Syn (Westphal and Chandra, 2013). α-Syn overexpression results in impaired membrane curvature and tubulation, which might be one of the mechanisms underlying the membrane disruption in PD (Varkey et al., 2010). Golgi fragmentation (Gosavi et al., 2002; Fujita et al., 2006), lysosome dysfunction (Meredith et al., 2002; Xilouri et al., 2016), mitochondrial degeneration (Martin et al., 2006; Wong and Krainc, 2017), and endoplasmic reticulum functional defects (Wong and Krainc, 2017) have been reported in cell lines and PD animal models with α-Syn overexpression. Both exocytosis and endocytosis are related to the membrane deforming. Due to the curvature-sensing and -forming property of α-Syn, it may participate in exocytosis, endocytosis and vesicle recycling physiologically and pathologically (Lautenschlager et al., 2017).

## α-Syn and Exocytosis

Neurotransmitter release from pre-synapse through exocytosis is crucial for efficient neuronal communication (Chapman, 2002; Sudhof, 2004; Wu et al., 2014). With the arrival of action potentials, extracellular calcium flows into the nerve terminals through voltage-gated calcium channels and triggers the soluble N-ethylmaleimide-sensitive factor attachment protein receptor (SNARE)-dependent vesicular exocytosis (Sudhof and Rothman, 2009; Rizo and Xu, 2015). SNARE complex, a key component for membrane fusion between vesicles and presynaptic terminals during neurotransmission (Weber et al., 1998), is composed of synaptobrevin (or vesicle associated membrane protein-2, VAMP2), syntaxin and synaptosome-associated protein 25 kD (SNAP25). VAMP2 is a synaptic vesicle protein (v-SNARE), whereas Syntaxin and SNAP25 (t-SNAREs) are localized in target membrane (Sollner et al., 1993). Each SNARE protein contains one (VAMP2 and syntaxin) or two (SNAP25) coiled-coil SNARE motifs, thus the assembly of t-SNARE and v-SNARE proteins forms a *trans*-SNARE complex containing a twisted parallel four-helix bundle that pulls the opposing membranes together and eventually initiates membrane fusion. Following exocytosis, the strained *trans*-SNARE complex relaxes into the fully zippered *cis*-SNARE, then is disassembled and recycled for another round of exocytosis (Sutton et al., 1998; Jahn and Scheller, 2006; Sudhof and Rothman, 2009). The molecular mechanism of SNARE-mediated membrane fusion has been summarized in recent reviews (Rizo and Xu, 2015; Baker and Hughson, 2016; Brunger et al., 2018; Tian et al., 2019). Here we mainly focus on the critical role of α-Syn in SNARE assembly and vesicular exocytosis.

**FIGURE 1:**
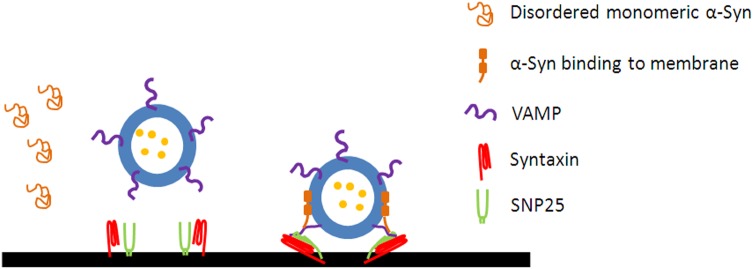
α-Syn promotes the assembly of SNARE complex. The disordered monomeric α-Syn in cytosol forms an amphipathic α-helix following the interaction with the membrane of vesicles. α-Syn binds to the N terminus of VAMP2 via its C terminus and to phospholipids via its N terminus to promotes the assembly of SNARE complex.

Exocytosis is divided into three sequential steps, docking, priming and vesicle fusion. Synaptic vesicles are tethered to specialized regions on the plasma membrane whereby they become stably bound. After docking, the vesicles are primed or matured through a series of ATP-dependent reactions. When an action potential invades, the voltage-gated Ca^2+^ channel-mediated calcium influx triggers the fusion pore opening of the primed vesicles (Sudhof, 1995). α-Syn participates all the three stages (Wang et al., 2016). (1) It is well-established that α-Syn facilitates the vesicles docking to the plasma membrane probably by increasing the SNARE complex assembly (Figure 1; Spillantini and Goedert, 2000; Burre et al., 2010, 2014; Georgieva et al., 2010; Lou et al., 2017). Interestingly, some studies show that α-Syn may function to bridge secretory vesicles and plasma membrane. One working model is that the N terminus of α-Syn binds to the plasma membrane and concurrently the C terminus interacts with VAMP2, thereby cross-bridging the plasma membrane and vesicle to facilitate the vesicle docking (Lou et al., 2017). The other model is that the broken alpha-helix conformation of α-Syn spanning between synaptic vesicles and the plasma membrane can also help to dock the vesicles to the plasma membrane (Georgieva et al., 2010). On the contrary, some studies show that α-Syn inhibits vesicle docking to the plasma membrane in a VAMP2-dependent or -independent manner. Specifically, larger α-Syn oligomers preferentially bind to the N terminus of VAMP2, inhibiting SNARE complex formation and thereby blocking the vesicle docking (Choi et al., 2013), which seems to contradict the fact that higher-order α-Syn multimers binds to membrane to promote SNARE complex formation (Burre et al., 2014). The divergence here needs to be clarified in the future study. Moreover, *in vitro* studies indicate that the inhibition is independent of VAMP2 (DeWitt and Rhoades, 2013) at the docking stage (Lai et al., 2014). (2) α-Syn is also involved in vesicle priming stage. The overexpression of WT or A30P α-Syn in PC-12 and chromaffine cells impairs catecholamine release probably by inhibiting vesicle priming. α-Syn overexpression increases the number of docked dense core vesicles, whereas the fusion machinery and the dynamics of amperometric quantal events remain unchanged (Larsen et al., 2006). (3) α-Syn is related to the dilation of fusion pore during vesicle fusion. α-Syn and other isoforms (β-Syn and γ-Syn) share the conserved N terminus, which is responsible for the pore dilation. Endogenous and overexpressed synuclein promote fusion pore dilation and cargo discharge, and slow down fusion pore re-closure in neurons and adrenal chromaffin cells (Logan et al., 2017). Moreover, PD-linked mutants, including A30P and A53T, which impair exocytosis, abrogate the property of α-Syn on pore dilation, suggesting that the mechanisms of α-Syn on release frequency and fusion pore dilation are different (Logan et al., 2017).

Collectively, α-Syn plays a critical role in the docking, priming, and fusion steps of exocytosis, probably by serving as a non-classical chaperone that facilitate SNARE assembly (Chandra et al., 2005; Burre et al., 2010, 2014). The mechanism by which α-Syn maintains the presynaptic SNARE complex assembly is the simultaneous binding of α-Syn to the N terminus of VAMP2 via its C terminus and to phospholipids via its N terminus (Burre et al., 2010). Whereas, only the membrane-bound α-Syn, which forms high-order multimers larger than octamers in a defined orientation, can function to chaperon SNARE complex assembly (Burre et al., 2014). It is proposed that α-Syn may also cooperate with cysteine string protein-α (CSPα) to maintain SNARE complex assembly. CSPα can form a chaperone complex with HSP70 (Heat-shock protein 70) and SGT (small glutamine-rich tetratricopeptide repeat-containing protein) to stabilize monomeric SNAP-25 and thus facilitate SNARE complex formation (Chamberlain and Burgoyne, 1997; Nemani et al., 2010). SNAP-25 undergoes deformation without the chaperone of CSPα, and subjects to ubiquitination and proteasome-dependent degradation, leading to the reduction of SNAP-25 and SNARE complex. CSPα knockout in mice leads to the reduction of SNAP-25 and SNARE complex, and thus the impaired exocytosis in pre-synapse. Overexpression of wild-type α-Syn mostly rescues CSPα deficiency-mediated neurodegeneration and SNARE complex assembly (Sharma et al., 2011), indicating a compensatory effect of α-Syn with CSPα in mediating SNARE protein assembly. Moreover, overexpression of α-Syn mutant A30P, but not A53T, fails to rescue the CSPα deficiency, indicating that the efficient binding to phospholipid is necessary for this compensating function of α-Syn (Chandra et al., 2005). However, not all of studies support the opinion that α-Syn facilitates SNARE complex assembly. Darios et al. report that there is no direct interaction between endogenous α-Syn and SNARE proteins (Darios et al., 2010). α-Syn may inhibit SNARE complex assembly and exocytosis by decreasing the levels of arachidonic acid (Darios et al., 2010), which is proposed to regulate SNARE complex formation and exocytosis in neurons and endocrine cells (Connell et al., 2007; Latham et al., 2007). Future research is needed to unveil the mechanisms underlying the divergence in the role of α-Syn in SNARE complex assembly and exocytosis.

## α-Syn and Endocytosis

To sustain the high rate of neurotransmission without depleting the recycling vesicle pools and to prevent the unlimited expansion of pre-synapse plasma membrane, endocytosis is tightly coupled to exocytosis to retrieve the fused synaptic vesicle components from the neuronal surface (Xie et al., 2017; Song et al., 2018). The tight coupling of endocytosis to exocytosis is critical for the maintenance of presynaptic structural integrity, the replenishing of the releasable vesicle pools and the sustained neurotransmission (Saheki and De Camilli, 2012; Wu et al., 2014; Leitz and Kavalali, 2016). α-Syn is reported to mediate membrane curvature and tabulation, a critical step in endocytosis. Clathrin-mediated endocytosis (CME) is the best characterized endocytic pathway, and is the predominant route for vesicle retrieval (McMahon and Boucrot, 2011). α-Syn mutants or overexpression impair endocytosis. In the calyx of held of A53T transgenic mice, both slow and fast vesicle endocytosis are inhibited (Xu et al., 2016). Furthermore, overexpression of human α-Syn induces a loss of synaptic vesicles and an expansion of the plasma membrane by inhibiting CME and activity-dependent bulk endocytosis. The phenomenon only occurs at 20 Hz, but not 5 Hz, stimulation, indicating that the defect is activity-dependent. Moreover, the N-terminal α-helix is also necessary for the regulatory role of α-Syn in endocytosis, as proved by PD-related mutant A30P with disrupted N-terminus (Busch et al., 2014). It is generally accepted that α-Syn oligomers co-exist with monomers under physiological conditions (Wang et al., 2014). Both monomers and oligomers impair CME, but with distinct mechanisms. Acute injection of α-Syn monomers into the lamprey reticulospinal synapse increases clathrin intermediates and clathrin-coated vesicles, indicating that the clathrin uncoating mechanism is impaired by α-Syn monomers. Whereas introduction of dimeric α-Syn increases clathrin-coated pits along the plasma membrane, suggesting a defect in vesicle fission (Medeiros et al., 2017). Furthermore, the inhibitory role of synuclein functions temporally before dynamin, indicating that synucleins act at early steps of vesicular endocytosis (Vargas et al., 2014). Along with endocytosis, receptor internalization is also mediated by α-Syn, i.e., α-Syn plays critical roles in regulating the homeostasis of dopamine transporter and NMDA receptor through CME (Cheng et al., 2011; Kisos et al., 2014).

## α-Syn and Vesicle Clustering

Endogenous α-Syn also promotes vesicle clustering (Diao et al., 2013; Fusco et al., 2016). By using a single-vesicle optical microscopy, Diao et al. (2013) show that α-Syn induces vesicle clustering, which is critically dependent on the interactions of α-Syn with VAMP2 and negatively charged lipids. Whereas only A30P mutant disrupts vesicles clustering due to the defects in lipid binding among three familial PD-related point mutants (A30P, E46K, A53T) (Diao et al., 2013). Another independent study shows that residues 1–25 of N terminus and residues 65–97 of central segment function as dual membrane anchors, by which α-Syn can simultaneously bind two different vesicles, thereby prompting vesicle clustering (Fusco et al., 2016). Physiologically, α-Syn regulates the trafficking of synaptic vesicles in distal reserve pool to control the amount of vesicles docked at the synapses for neurotransmitter release (Wislet-Gendebien et al., 2006; Auluck et al., 2010). The physiological role of α-Syn is to bind to and induce the clustering of synaptic vesicles *in vitro* and *in vivo* (Kwasny et al., 1991; Gitler et al., 2008; Soper et al., 2008; Diao et al., 2013). In cultured hippocampal slices, α-Syn knockdown decreases the distal reserve synaptic vesicle pool by 50% with the intact docked vesicle pool (Murphy et al., 2000). Modest overexpression of α-Syn in hippocampal neurons displays a reduction in the number of docked vesicles and increase in the number of vesicles in the distal reserve pool, indicating a defect in the re-clustering of synaptic vesicles after endocytosis (Nemani et al., 2010).

## Conclusion

**FIGURE 2:**
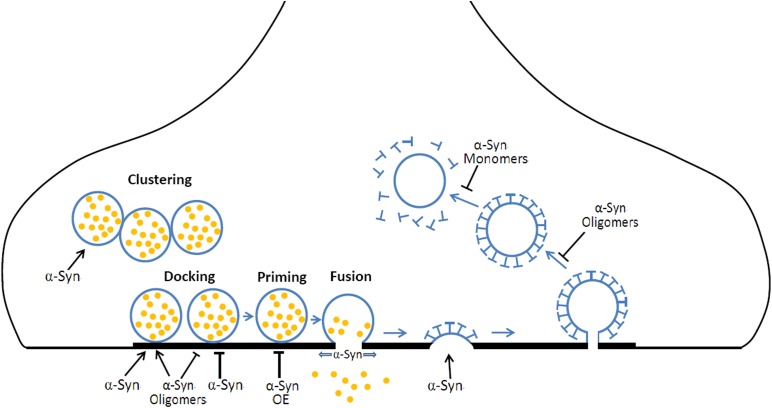
The roles of α-Syn in vesicular exocytosis and endocytosis. During neurotransmission, (1) α-Syn mediates the clustering of synaptic vesicles, resulting in a local increase of releasable vesicles. (2) α-Syn and α-Syn oligomers prompt or inhibit vesicle docking at the active zone. (3) α-Syn overexpression (OE) inhibits the priming of synaptic vesicles. (4) α-Syn promotes the fusion pore opening and slows fusion pore reclosing. (5) α-Syn is required for CME at early steps. (6) α-Syn oligomers OE results in a defect in vesicle fission while α-Syn monomers OE induces a defect in uncoating mechanism during CME.

α-synuclein is a membrane curvature sensing and deforming protein, functioning in exocytosis and endocytosis (Figure 2). The exact roles of α-Syn are still ambiguous, far from understood and still need to be clarified for a better understanding of the physiological and pathological roles of α-Syn. Moreover, the deficiency in exocytosis, endocytosis, and vesicles recycling may underlie a common pathogenic pathway shared by other PD risky genes (i.e., *synaptotagmin-11*, *parkin*, *LRRK2*, *DJ-1*, *synaptojanin1*, and *TMEM230*) (Abeliovich and Gitler, 2016; Pan et al., 2018; Wang et al., 2018). Advances in super resolution microscopy, single-molecule FRET imaging, cryo electron microscopy offer new opportunities for further studies about the physiological role of α-Syn in exocytosis, endocytosis, and vesicle recycling, while *in vivo* genetic modulation, optogenetic/chemical genetic stimulation, *in vivo* optometric recording or two photon imaging will benefit the pathogenic connections between α-Syn, vesicle recycling, and neurodegeneration. It will reveal novel therapeutic targets for PD in gaining insight into the mechanisms of these PD risk genes in exocytosis, endocytosis, and vesicles recycling.

## Author Contributions

MH, BW, and XL drafted the manuscript with help from CF, CW, and XK. All authors coordinated, revised, and approved the manuscript.

## Conflict of Interest Statement

The authors declare that the research was conducted in the absence of any commercial or financial relationships that could be construed as a potential conflict of interest.
